# ON and OFF receptive field processing in the presence of optical scattering

**DOI:** 10.1364/BOE.489117

**Published:** 2023-05-11

**Authors:** Katharina Breher, Antonia Neumann, Dominik Kurth, Frank Schaeffel, Siegfried Wahl

**Affiliations:** 1 Carl Zeiss Vision International GmbH, Turnstr. 27, 73430 Aalen, Germany; 2 Institute for Ophthalmic Research, University of Tübingen, Elfriede-Aulhorn-Str. 7, 72076 Tübingen, Germany; 3 Institute of Molecular and Clinical Ophthalmology Basel, Mittlere Str. 91, 4056 Basel, Switzerland

## Abstract

The balance of ON/OFF pathway activation in the retina plays a role in emmetropization. A new myopia control lens design uses contrast reduction to down-regulate a hypothesized enhanced ON contrast sensitivity in myopes. The study thus examined ON/OFF receptive field processing in myopes and non-myopes and the impact of contrast reduction. A psychophysical approach was used to measure the combined retinal-cortical output in the form of low-level ON and OFF contrast sensitivity with and without contrast reduction in 22 participants. ON responses were lower than OFF responses (ON 1.25 ± 0.03 vs. OFF 1.39 ± 0.03 log(CS); p < 0.0001) and myopes showed generally reduced sensitivities (myopes 1.25 ± 0.05 vs. non-myopes 1.39 ± 0.05 log(CS); p = 0.05). These findings remained unaffected by contrast reduction (p > 0.05). The study suggests that perceptual differences in ON and OFF signal processing between myopes and non-myopes exist but cannot explain how contrast reduction can inhibit myopia development.

## 1. Introduction

In the vertebrate retina, retinal ganglion cells (RGCs) are arranged in specific structures forming receptive fields. The receptive field of a single cell is defined as the restricted region in space that the cell responds to with excitation or inhibition [1]. Regarding the RGCs, receptive fields are the smallest in the central retina and the largest in the peripheral retina [2]. One of the basic distinctions that can be made between receptive fields is that they can have either "ON" or "OFF" structure. The ON center receptive fields fire in response to a bright stimulus in the center and a dark surround region whereas the OFF center receptive fields work vice versa [3]. Therefore, receptive field processing presents a low-level operation to permit contrast vision.

Contrast processing is assumed to have an influence on myopia development [4–6]. However, differing hypotheses were established with regards to low-level contrast processing. In the past, a deficiency of the ON pathways was shown to be related to myopia in human retinopathy of prematurity, potentially via a dopaminergic mechanism [7]. Furthermore, choroidal thickening - viewed as anti-myopiagenic, as it is associated with slower eye growth - was observed with ON pathway stimulation in another human study [4,8]. Similar conclusions were drawn from a mouse model with mutations causing defect ON-pathways and a subsequently higher susceptibility to myopia [9].

Clearly conflicting, a hypothesis linking receptive field processing and myopia was established based on the hereditary Bornholm eye disease [10]. Here, the lack of glutamate, which is necessary to inhibit ON bipolar cells, leads to a constantly higher activation and contrast signaling of these cells. This thought process is supported by genetic mutations that produce malfunctioning neighboring cones that also lead to an intensified contrast signaling, especially of the ON pathways. It is assumed that the up-modulated ON signaling stimulates axial elongation in affected individuals [10–13]. These novel hypotheses are in line with a study in kittens, where pharmacological ON channel blockage was associated with more hyperopia [14].

Despite the discrepancies between the aforementioned studies, all findings have in common that they point towards an important role of the ON pathways in the emmetropization process [15].

Following the novel assumption that over-activation of ON pathways may stimulate myopia, spectacle lenses were developed to reduce image contrast in the retina via optical scattering. An early clinical trial showed promising effects on myopia of this type of lens design: progression of axial length and refractive error were reduced by 0.15 mm and 0.40 D compared to a control lens [13]. However, the results of this clinical trial conflict with evidence from animal models that retinal image degradation induces form-deprivation myopia [16]. Furthermore, the effects of strong contrast reduction on axial length and choroidal thickness in humans were like those of negative defocus [17]. Therefore, the working mechanism of contrast reduction on visual processing and myopia development are not clarified yet.

In summary, hypotheses regarding the potential effects of optical scattering on receptive field processing and myopia development remain conflicting and unclear. According to the rationale for the design of contrast-reducing spectacle lenses, especially the ON pathway is up-regulated in myopes due to abnormally high retinal contrast signaling. The over-stimulation is suggested to be attenuated when optical scattering is applied. Therefore, the purpose of this study was to compare the effects of optical scattering on low-level ON and OFF contrast processing in myopic and non-myopic subjects.

## 2. Materials & methods

### 2.1 Study participants

The study was approved by the ethics committee of the medical faculty of the University of Tübingen. Before the start of the measurements, the study procedures were explained to the participants, who then gave their written informed consent. All participants were of self-reported good ocular health. In addition, screening for refractive-corneal and retinal pathologies was performed using wavefront aberrometry (ZEISS i.Profiler+, Carl Zeiss Vision GmbH, Aalen, Germany) and optical coherence tomography (ZEISS PlexElite 9000, Carl Zeiss Meditec Inc., Dublin, USA), respectively.

A total of 22 subjects participated in the study. The number of myopes (≤ -0.5 D) and non-myopes (> -0.5 D) were matched in two groups of each n = 11. The myopic group had a median spherical equivalent refractive error of −2.75 D (range −0.50 D to −9.00 D), while the non-myopic group showed a distribution of 0.00 D (range −0.38 D to +1.13 D). It was sought to keep the age and gender composition of both groups similar (myopes 26 ± 3 years vs. non-myopes 25 ± 4 years; p = 0.61), with 7 males and 4 females in the myopic and 6 males and 5 females in the non-myopic group.

### 2.2 Test setup and instrumentation

Subjective refraction of the studied right eye was measured using the ZEISS VISUSCREEN 500 and ZEISS VISUPHOR 500 digital phoropter (Carl Zeiss Vision GmbH, Aalen, Germany). Subsequently, the spherical and astigmatic refractive errors were corrected with a trial frame (Oculus B5, Oculus GmbH, Wetzlar, Germany) and trial lenses (Oculus BK 1/T, Oculus GmbH, Wetzlar, Germany) in a vertex distance of 12 mm. In the conditions with optical scattering, a 0.6 Bangerter foil (Breitfeld & Schliekert GmbH, Karben, Germany) was added to the trial lenses, which reduced visual acuity by 0.2 logMAR. The left eye was covered with an infrared filter allowing to track fixation by an eye tracker. The EyeLink 1000 plus (SR Research, Ottawa, Ontario, Canada) was utilized with an infrared-based eye tracking frequency of 1000 Hz. The eye tracker was located beneath the participant’s left eye at 30 cm distance and an individual calibration was carried out before the actual experimental measurements.

The computer running the psychophysical test was equipped with an Nvidia Geforce 1080GTX graphics card and an i7 processor. For stimulus presentation, a VIEWPixx display (VPixx Technologies Inc., Saint-Bruno, QC, Canada) with a resolution of 1920 x 1200 pixels and a gamma correction factor of 2.02 was used. Monitor luminance was 40 cd/m^2^, room illumination was fixed at 25 lux. The monitor was located at 98 cm distance and centered to the participant’s right eye. The experimental test was programmed in MATLAB R2020b (MathWorks Inc., Natick, Massachusetts, USA) in combination with the Psychtoolbox [18], Palamedes toolbox [19] and the DataPixx toolbox (VPixx Technologies Inc., Saint-Bruno, QC, Canada).

### 2.3 Stimulus design and experimental procedure

**Fig. 1.**
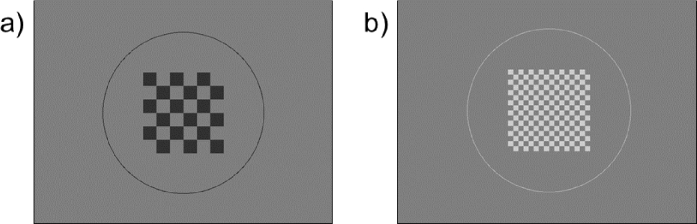
Examples of stimuli for (a) OFF polarity at 1.5 cpd and (b) ON polarity at 6 cpd.

A white/grey checkerboard (ON polarity) and a black/grey checkerboard (OFF polarity) on uniform grey background were displayed to measure low-level contrast sensitivity, as seen in Fig. 1. The stimulus covered a visual angle of around 1.7° and was adjusted for magnification effects caused by the participant’s trial lenses for refractive correction. The number of checkerboard fields varied while the total stimulus size remained the same to test different spatial frequencies, also shown in Fig. 1. Spatial frequencies were calculated as previously suggested for checkerboard stimuli by Iyer et al. [20].

The selected spatial frequencies were 1.5, 3, 6 and 12 cpd. Stimulus luminance averaged over all spatial frequencies at full contrast was 65 ± 1 cd/m^2^ for the ON pattern and 23 ± 1 cd/m^2^ for the OFF pattern. In total, the low-level contrast sensitivity was tested for two contrast polarities (ON vs. OFF), two retinal locations (central 0° vs. temporal 10°) and two scattering conditions (with vs. without scattering). To maintain the attention of the subjects, the experiment was performed at two separate days: one day where all conditions were tested without scattering lens in place and one day with the scattering lens in place. The order of the testing paradigms was randomized for each participant and test day to avoid biases caused by adaptation effects or fatigue.

**Fig. 2.**
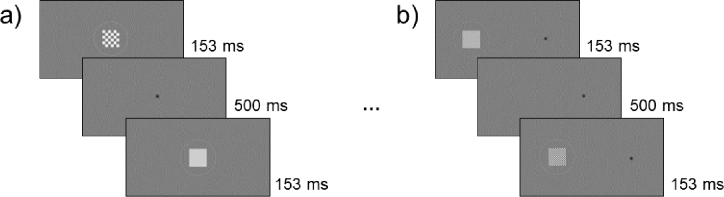
Example of a trial to test the ON condition at (a) central testing with 1.5 cpd and at (b) peripheral testing with 6 cpd.

The experiment followed a two-interval forced-choice (2IFC) paradigm, where only one of two shown screen frames contained the checkerboard stimulus, separated by a blank grey screen. To preserve the attention of the participants, both 2IFC screens contained an additional ring around the stimulus with the same contrast polarity as the checkerboard stimulus, while the blank area in between facilitated maintained fixation. After each trial, a grey screen appeared while the participant indicated via keypress in which frame the stimulus was perceived. Both stimulus frames were shown for 153 ms and the blank grey screen in between for 500 ms. The non-checkerboard frame showed a same-sized square with half the contrast to the background compared to the checkerboard stimulus contrast to equalize luminance between both screens. This implementation was made to ensure that the participant was not guided by luminance differences between screen frames. The low-level contrast sensitivity measurement for each spatial frequency measurement consisted of 30 trials within the PSI adaptive staircase method. The procedure of a central and peripheral measurement session is exemplified in Fig. 2.

To reduce the impact of fluctuations in accommodation, a lens of +1 D was added to the refractive correction of the participant. Contrast sensitivity was assessed for the right eye, while eye tracking was performed on the left eye, which was covered by an infrared filter. The left eye was tracked with an eye tracker to verify correct fixation during the peripheral testing. Stimulus presentation was paused for 500 ms and restarted if the eye tracker detected blinks or deviating fixation by more than 3.8°.

### 2.4 Statistical analysis

Data analysis was performed using MATLAB R2020b (MathWorks Inc., Natick, Massachusetts, USA) and JMP (JMP 16, SAS Institute Inc., Cary, North Carolina, USA). The measured low-level contrast sensitivity thresholds were converted to low-level log(CS) and outlier data points outside the range of three standard deviations from the mean were excluded from analysis. Normal distribution of the sample data was verified with the Lilliefors test.

To identify significant factors on low-level log(CS) as dependent variable, a mixed model analysis was performed. The model was fitted using refractive group (2 levels), spatial frequency (4 levels), pattern polarity (2 levels), retinal location (2 levels) and scattering presence (2 levels) as fixed effects. Refractive group was additionally nested within "subjects", which was set as random effect, to represent the repeated-measures and between-subject character of the study. Subsequently, the model linearity and normality of the residuals were visually confirmed. Significant model factors with p ≤ 0.05 underwent multiple comparisons using Tukey’s test and are reported as least-squares means ± standard error. Data pooling was performed while controlling for the individual model factors using estimated marginal means. Only model terms of interest and statistical significance are reported in the results section to maintain clarity due to the high number of possible interactions between parameters.

## 3. Results

**Fig. 3.**
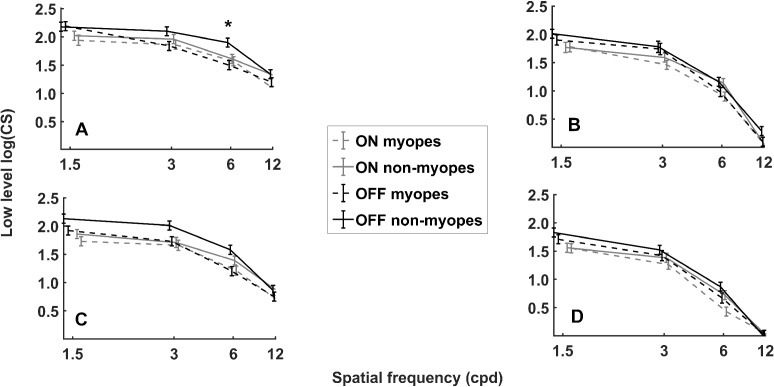
Contrast sensitivity functions (least-squares mean ± standard error, n = 22 participants) for (a) central testing without scattering; (b) temporal 10° testing without scattering; (c) central testing with scattering and (d) temporal 10° testing with scattering. Asteriks denote significant differences between refractive groups for the same testing condition (* p < 0.05).

Mean low-level contrast sensitivities are presented in Fig. 3 and Table 1–4, as well as in the Supplement 1 with individual data points (see Figure S1).

**Table 1.** Low-level log(CS) in the central measurement conditions for n = 11 myopes (least-squares mean ± standard error).

	**Without scattering**		**With scattering**	
	ON	OFF	ON	OFF
**1.5 cpd**	1.94 ± 0.09	2.19 ± 0.08	1.73 ± 0.08	1.92 ± 0.08
**3 cpd**	1.86 ± 0.08	1.84 ± 0.08	1.66 ± 0.09	1.73 ± 0.08
**6 cpd**	1.57 ± 0.08	1.50 ± 0.08	1.21 ± 0.08	1.20 ± 0.08
**12 cpd**	1.07 ± 0.08	1.20 ± 0.08	0.69 ± 0.08	0.75 ± 0.08

**Table 2.** Low-level log(CS) in the central measurement conditions for n = 11 non-myopes (least-squares mean ± standard error).

	**Without scattering**		**With scattering**	
	ON	OFF	ON	OFF
**1.5 cpd**	2.02 ± 0.08	2.18 ± 0.08	1.86 ± 0.08	2.13 ± 0.08
**3 cpd**	1.96 ± 0.08	2.10 ± 0.08	1.71 ± 0.09	2.01 ± 0.08
**6 cpd**	1.61 ± 0.08	1.90 ± 0.08	1.39 ± 0.08	1.58 ± 0.08
**12 cpd**	1.32 ± 0.08	1.34 ± 0.08	0.86 ± 0.08	0.88 ± 0.09

**Table 3.** Low-level log(CS) in the temporal measurement conditions for n = 11 myopes (least-squares mean ± standard error).

	**Without scattering**		**With scattering**	
	ON	OFF	ON	OFF
**1.5 cpd**	1.78 ± 0.09	1.90 ± 0.09	1.55 ± 0.08	1.71 ± 0.08
**3 cpd**	1.46 ± 0.08	1.74 ± 0.10	1.26 ± 0.08	1.42 ± 0.09
**6 cpd**	0.90 ± 0.08	0.98 ± 0.08	0.43 ± 0.08	0.67 ± 0.09
**12 cpd**	0.01 ± 0.09	0.10 ± 0.08	0.02 ± 0.08	0.02 ± 0.08

**Table 4.** Low-level log(CS) in the temporal measurement conditions for n = 11 non-myopes (least-squares mean ± standard error).

	**Without scattering**		**With scattering**	
	ON	OFF	ON	OFF
**1.5 cpd**	1.77 ± 0.09	2.01 ± 0.08	1.56 ± 0.08	1.83 ± 0.08
**3 cpd**	1.59 ± 0.08	1.78 ± 0.10	1.38 ± 0.08	1.52 ± 0.08
**6 cpd**	1.14 ± 0.08	1.16 ± 0.08	0.72 ± 0.08	0.87 ± 0.08
**12 cpd**	0.03 ± 0.09	0.29 ± 0.08	0.00 ± 0.08	0.00 ± 0.09

Spatial frequency, optical scattering and testing location were shown to be highly significant factors in the model with all p < 0.0001 on their own and in interactions between them. As these findings were expected, they will not be detailed further here.

Pattern polarity had a significant effect in the model, where contrast sensitivity with OFF patterns was higher than with ON patterns (OFF 1.39 ± 0.03 vs. ON 1.25 ± 0.03 log(CS); p < 0.0001). Interestingly, also refraction had a borderline significant effect: averaged over all test conditions, myopes showed reduced low-level sensitivities compared to non-myopes (myopes 1.25 ± 0.05 vs. non-myopes 1.39 ± 0.05 log(CS); p = 0.05).

A significant two-factorial interaction was refractive group in combination with spatial frequency (p = 0.01), where myopes and non-myopes showed significantly different sensitivities at 6 cpd with 1.06 ± 0.05 and 1.30 ± 0.05 log(CS) (p = 0.03), respectively. Moreover, pattern polarity in combination with spatial frequency also showed an statistically significant influence on contrast sensitivity (p = 0.04), with ON and OFF sensitivities being significantly different for all spatial frequencies except 12 cpd (1.5 cpd: ON 1.77 ± 0.04 vs. OFF 1.98 ± 0.04 log(CS), p < 0.0001; 3 cpd: ON 1.61 ± 0.04 vs. OFF 1.77 ± 0.04 log(CS), p = 0.001; 6 cpd: ON 1.12 ± 0.04 vs. OFF 1.23 ± 0.04 log(CS), p = 0.03).

The last statistically significant interaction term of interest was between refraction group, pattern polarity, retinal location and spatial frequency with p = 0.02. Here, a difference between myopes and non-myopes was found for OFF low-level sensitivities at the central 6 cpd testing condition (myopes 1.36 ± 0.07 vs. non-myopes 1.74 ± 0.07 log(CS); p = 0.02).

Besides the expected reduction of low-level contrast sensitivity with optical scattering, no further interactions with pattern polarity (OFF/ON) or refraction (myopes/non-myopes) could be detected (all p > 0.05).

## 4. Discussion

The study was performed to investigate the impact of optical scattering on receptive field processing in myopes and non-myopes at two retinal locations. All investigated factors had a significant influence on their own on low-level contrast sensitivity. Low-level log(CS) declined significantly with increasing spatial frequency, in the retinal periphery, with induced optical scattering, as well as in myopes and with ON pattern polarity compared to non-myopes and OFF polarity. Besides its plausible individual influence, optical scattering had no significant impact on receptive field processing in combination with pattern polarity or refractive state. Myopes neither exhibited an enhanced ON sensitivity compared to non-myopes nor had optical scattering an asymmetrical influence on ON/OFF pathway processing concerning refractive error. This contradicts the rationale for the design of contrast-reducing spectacle lenses for myopia control, which is supposed to attenuate an enhanced ON sensitivity in myopes as the cause for myopia progression.

### 4.1 Interpretation of ON and OFF contrast sensitivity curves

It is also noteworthy, that both ON and OFF contrast sensitivity functions peak at 1.5 cpd compared to usual peaks between 2 cpd and 5 cpd [21]. This effect could be caused by the checkerboard stimulus itself. In the case of checkerboard stimuli, a more recent study found VEP peak sensitivities between 1.5 cpd and 3 cpd as well [20].

Moreover, the current study revealed a general difference in ON and OFF receptive field processing. Here, the low-level OFF responses were significantly higher than the low-level ON responses. Humans perceive dark stimuli faster and more accurately compared to bright stimuli due to the functional and structural differences of the ON and OFF pathways [22–27]. An explanation for this phenomenon is the dominance of the OFF visual pathway in the primary visual cortex and a higher number of OFF bipolar cells, especially in the center of the retina, except directly in the fovea [24,28,29]. These physiological features in combination with the relatively short stimulation time of 153 ms may have caused the observed differences. Moreover, the OFF dominance depends on spatial frequency content, caused by a different spatial structure and processing specialization of the ON and OFF cells [27,28]. For example, Jansen et al. [28] showed a larger ON vs. OFF difference for lower spatial frequencies compared to the high spatial frequency range, which is in line with the presented findings. ON pathways also have larger receptive fields, smaller dynamic range and slower responses than OFF pathways. Altogether, this could have affected OFF pathways more than ON pathways in the current study [27,28].

### 4.2 ON and OFF processing in the context of myopia

In the context of myopia, this study detected differences between refractive groups in low-level contrast sensitivity processing. However, the current study showed an absolute reduction - instead of up-regulation - of sensitivities in myopes, as already found previously for conventional contrast sensitivity [6]. Moreover, there was a tendency of stronger differences between refractive groups for OFF than ON responses, most pronounced centrally at 6 cpd. No significant changes in processing were induced by scattering at the peripheral testing location. Therefore, our findings do not help to explain why peripheral scattering should inhibit myopia development [13].

Ergo, the relation between ON/OFF receptive field processing and myopia development remains unclear. Wang et al. showed in a study with chickens that dynamic ON stimuli led to thicker choroids, while OFF stimuli caused choroidal thinning [30], which was assumed to predict future eye growth [31,32]. Moreover, Aleman et al. found bi-directional effects on choroidal thickness with ON or OFF stimulation in young human subjects. Black text on white background, mainly stimulating the OFF channels, led to a thinning of the choroid within an hour of exposure. In contrast, white text on black background for ON channel stimulation resulted in choroidal thickening [4]. Recently, a study in humans also showed that reading, which is associated with myopia progression, is under-stimulating the ON pathways [33]. These previous findings on protective effects of relative ON channel activation are in line with refractive development in human retinopathy of prematurity: here, reduced ON pathway activity was shown to disrupt emmetropization and led to a significantly higher risk of developing myopia [7]. The same relation accounts for genetically modified mice with deficient/defect ON pathways that present with a higher susceptibility to myopia [9].

The role of central vs. peripheral or cone vs. rod-mediated ON and OFF pathway activity is also not fully clarified yet. The 10° temporal stimulus is not fully representative for rod and peripheral processes. As the primate retina only consists of ON rod bipolar cells, it would be expected that the OFF responses would decline with increasing retinal eccentricity. Interestingly, Wan et al. found increased rod responses in myopia with full-field light and dark ERG recordings, which suggests that ON/OFF processing varies across the retina [34].

Furthermore, the influence of chromatic signals from the different cones still needs to be researched in more detail. With cone-specific chromatic stimuli, absolute ON and OFF sensitivity are expected to be lowest for the S-cones, as shown before with general contrast sensitivity and ERG responses [35–37]. Additionally, the relation between OFF and ON sensitivity is suggested to remain similar as all cones are connected to ON and OFF cells. In the myopic retina, the contrast theory from the myopia control lens suggests that the L- and M-cones are most affected by mutations, which will eventually increase the ON activity. This means that stronger effects could be visible in future studies using L/M-cone stimuli.

Moreover, contrast adaptation as a more long-term parameter in response to altered image quality (by defocus or scattering) seems to be a major regulator of eye growth [32,38]. There is an ongoing debate around optical scattering traditionally being used as a means to induce form-deprivation myopia vs. being applied in novel myopia control lenses as conflicting perspectives. Smith and Hung showed in monkeys that the degree of image degradation correlates with the amount of form-deprivation myopia [2]. It may be only subtle differences in parameter composition - for example the degree and position of contrast reduction, size of the clear central zone or size of the scatter elements - that determine whether optical scattering figures as an anti-myopiagenic or a myopiagenic tool.

### 4.3 How representative are psychophysical measurements of ON/OFF contrast sensitivity for retinal mechanisms of emmetropization?

There is abundant evidence that emmetropization is controlled by the retina, with no convincing evidence of input from the brain [32]. In previous studies supporting a role of the retinal ON/OFF system in myopia development, either changes in choroidal thickness were measured (i.e. chickens: [30,39]; humans: [4,40,41]) or long-term changes in eye growth (chickens: [42,43]). These studies therefore really measured retinal control of growth of the underlying tissues. This was different in the current study where a psychophysical approach was used, and the combined retinal and cortical output was measured. Even though we cannot trace the observed differences in low-level contrast processing in non-myopes and myopes solely down to the retina, the finding that such differences exist is important. Even more important, this relationship was not influenced by optical scattering, showing that scattering cannot make myopes more like non-myopes. The small differences in ON/OFF processing may not affect general visual performance and one could raise the hypothesis that they originate in the retina and may be specifically related to retinal signals driving myopia development. On the other hand, it cannot be excluded that conscious perception of contrast is completely separate from emmetropization and uses different retinal ganglion cells. In this case, we would not be able to learn about retinal mechanisms for emmetropization.

Electrophysiology can provide more insights into direct retinal mechanisms than psychophysical procedures. Previous research found generally reduced/delayed responses with myopia with a focus on photoreceptor activity [44]. Another study primarily showed reduced combined ON/OFF responses in myopes [45]. It is possible, while not trivial and without limitations, to extract separate ON and OFF responses from suitable ERG protocols [46]. To the authors’ best knowledge, only one study investigated separate ON vs. OFF electrophysiology responses in relation to myopia, who, however, had a history of retinopathy of prematurity [7]. Here they found that the reduction of ON and OFF activity is associated with the degree of myopia. Future electrophysiology studies would be therefore fruitful to add information to the current psychophysical results.

### 4.4 Potentially confounding effects explaining the asymmetry of ON vs. OFF contrast sensitivity

The low-level contrast sensitivity was determined by psychophysics and therefore does not only reflect retinal but also cortical functions. Emmetropization is assumed to be controlled by local retinal processes, which could not be extracted here. Moreover, the current study comprised young adults with stable low to moderate myopia and one participant with high myopia. In combination with a relatively small sample size and an inhomogeneous distribution of refractive errors, this did not allow clustering of different degrees of myopia to investigate in more detail. It is possible that higher degrees of myopia show more distinct effects, especially with high myopes as shown for other visual functions [47–50]. Therefore, the results might not be directly representative for children with progressing myopia as were investigated by Rappon et al. [13].

Due to shifts in fixation and attention, there is the possibility of participants being non-purposely exposed to the opposite polarity of the stimulus. In other words, participants fixating onto a grey area in the OFF stimulus would lead to a stimulation of the ON pathway instead. To prevent such biases as best as possible, no grey areas were placed directly in the center of the stimulus (targeted fixation point). Moreover, the general appearance of the stimulus outside the distinct fixation location showed a clearly defined polarity in its entirety, comparable to the text stimulus by Aleman et al. [4]. The stimulus time in this study was limited to 153 ms. Since in humans, the perception of dark stimuli is faster compared to bright stimuli [24,27], an extended stimulus presentation time might lead to different results regarding the discrepancy of ON and OFF log(CS) values.

Pupil size was not monitored and therefore, influences from ray vergence, cell crosstalk and/or effective retinal illuminance cannot be fully excluded [51]. However, similar pupil sizes can be assumed due to the sample composition (similar age and ethnicity) and test conditions (room illumination). The lack of a strong effect of optical scattering could be caused by the short-term wear of the filters but also by the used filters themselves, which were Bangerter foils in the current study. The modulation transfer function of a 0.6 Bangerter foil suggests a relative contrast reduction of 0.3 for 1.5 cpd, 0.65 for 3 cpd and approximately 0.9 for 6 cpd and 12 cpd [52]. Pérez et al. also mentioned manufactural inconsistencies in the physical structure and optical properties of different Bangerter foil densities [52]. This was controlled by using the same foil for all participants. Even though the underlying manufacturing technology of scattering probably differs between the novel myopia control lenses and conventional Bangerter foils [53], both types consist of circular protuberances that diffuse the light, thus, reducing visual acuity and contrast. Moreover, the myopia control lenses reduce visual acuity by approx. 1 line, whereas the 0.6 Bangerter foil reduces visual acuity by approx. 2 lines on the vision chart. The 0.6 Bangerter foil was preferred to ensure a reliable reduction of visual acuity by at least 1 line, which was not always achieved with the 0.8 Bangerter foil, while still maintaining high comparability to the myopia control lenses.

## 5. Conclusion

We investigated the influence of optical scattering on receptive field processing in myopes and non-myopes using a psychophysical procedure. Low-level contrast sensitivities tend to be reduced in myopes compared to non-myopes and this effect persisted independent of the presence of optical scattering. These findings do not support the hypothesis that was proposed to explain why optical scattering should inhibit myopia, acting as an equalizer of receptive field processing. The proposal that scattering should inhibit myopia, rather than stimulating it, contrasts with the classical concept of deprivation myopia.

## Data Availability

Data underlying the results presented in this paper are not publicly available at this time but may be obtained from the authors upon reasonable request.

## Supplemental document

See Supplement 1 for supporting content.
