# Supplementary material for: ON and OFF receptive field processing in the presence of optical scattering
Source: Biomed Opt Express. 2023 May 11;14(6):2618–28. doi: 10.1364/BOE.489117 (PMC10278613; doi:10.1364/BOE.489117)
Supplement: Supplementary file 1 [file boe-14-6-2618-s001.pdf]

Supplemental Document

Biomedical Optics EXPRESS

ON and OFF receptive field processing in the presence of optical scattering: supplement

KATHARINA BREHER,[1,2,*] ANTONIA NEUMANN,[2] DOMINIK KURTH,[2] FRANK SCHAEFFEL,[2,3] AND SIEGFRIED WAHL[1,2]

[1] *Carl Zeiss Vision International GmbH, Turnstr. 27, 73430 Aalen, Germany*
[2] *Institute for Ophthalmic Research, University of Tübingen, Elfriede-Aulhorn-Str. 7, 72076 Tübingen, Germany*
[3] *Institute of Molecular and Clinical Ophthalmology Basel, Mittlere Str. 91, 4056 Basel, Switzerland*
[*] *katharina.breher@uni-tuebingen.de*

This supplement published with Optica Publishing Group on 11 May 2023 by The Authors under the terms of the Creative Commons Attribution 4.0 License in the format provided by the authors and unedited. Further distribution of this work must maintain attribution to the author(s) and the published article's title, journal citation, and DOI.

Supplement DOI: https://doi.org/10.6084/m9.figshare.22643410

Parent Article DOI: https://doi.org/10.1364/BOE.489117

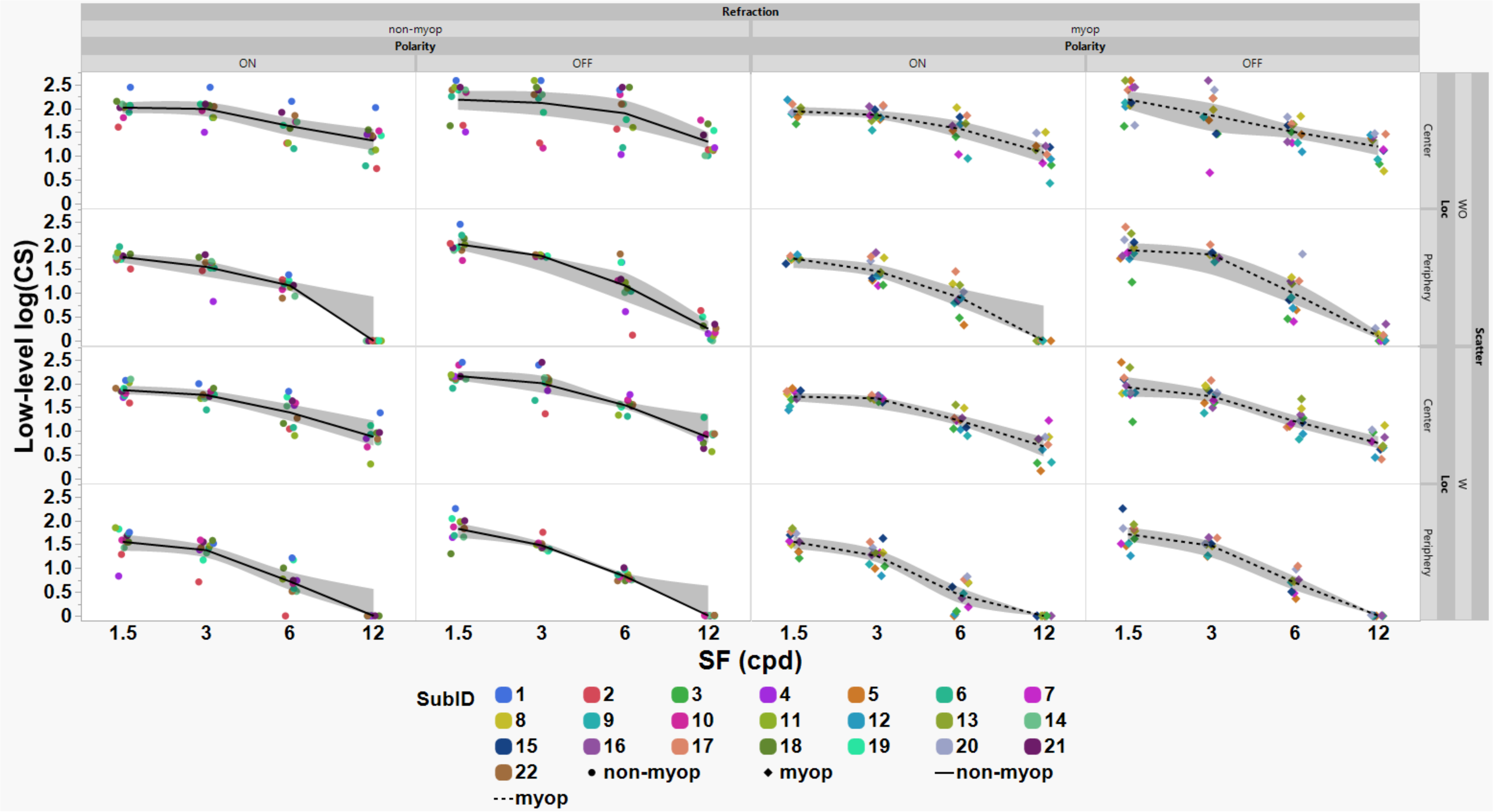

Figure S1: Visualization of the results from Figure 3 but with individual data points.
